# POSAiDA: presence of *Staphylococcus aureus*/MRSA and *Enterococcus*/VRE in Danish ambulances. A cross-sectional study

**DOI:** 10.1186/s13104-016-1982-x

**Published:** 2016-03-30

**Authors:** Heidi Storm Vikke, Matthias Giebner

**Affiliations:** Section of Research and Development, Medical Department, Falck Denmark A/S, Caspar Müllers Gade 2, 6000 Kolding, Denmark; Medical Department, Falck Denmark A/S, Kolding, Denmark

**Keywords:** Hygiene, Contamination, Infection control, Cleaning procedures, Prehospital, Emergency care

## Abstract

**Background:**

Every year approximately one out of ten Danish patients contracts a healthcare associated infection (HAI). *Staphylococcus aureus* and *Enterococcus* are prominent in the group of pathogenic bacteria that underlie HAIs, causing unnecessary inconvenience and prolonging hospitalization. Bacterial colonization often occurs due to indirect patient-to-patient transmission, caused by poor hygiene compliance. This study aims to determine the level of contamination with *S. aureus*/MRSA and *Enterococcus*/VRE on presumed clean blood pressure cuffs in the Danish ambulances.

**Method:**

Blood pressure cuffs were tested for contamination with *S. aureus* and *Enterococcus* when being cleaned according to everyday guidelines in this cross-sectional study. Imprints were performed with specific agar plates after cleaning with ethanol wipes. Positive imprints were typed and antibiotic susceptibility was determined.

**Results:**

Both *S. aureus* and *Enterococcus* were found on blood pressure cuffs thought to be clean, however, to a limited extent. The average level of contamination by *S. aureus* was 0.54 CFU per 25 cm^2^ (SD 1.98). Minimum and maximum values ranged from 0 to 12 CFU per 25 cm^2^ and 10 % of the 50 samples were positive. The average level of contamination by *Enterococcus* was 0.06 CFU per 25 cm^2^ (SD 0.42). Minimum and maximum values ranged from 0 to 3 CFU per 25 cm^2^ and 2 % of the 50 samples were positive. All *S. aureus* isolates were found to be methicillin susceptible *S. aureus* (MSSA) and the one *Enterococcus* isolate was identified as *Enterococcus faecalis*, negative for vancomycin resistance genes.

**Conclusion:**

*Staphylococcus aureus* and *Enterococcus* were detectable on equipment thought to be clean. However, all detected bacteria showed susceptibility towards methicillin or vancomycin. Findings of pathogens after cleaning may be due to cross-contamination, improper cleaning and limited effect of the currently used cleaning procedure and are thought to affect the risk of infection. Therefore, we recommend a thorough evaluation of current cleaning procedures as well as increased focus on and further research into hygiene challenges in a prehospital setting. Future studies should be performed in order to demonstrate the level of bacterial contamination in all areas of the medical service, e.g., the ambulance environment, medical equipment, staff uniform and hand hygiene. Furthermore, in order to establish evidence for different cleaning procedures in situ we recommend testing the effect of different cleaning interventions by interventional designs.

## Background

Every year approximately one out of ten Danish patients contracts an infection during their hospitalization [1]. *Staphylococcus aureus* and *Enterococcus* are prominent in the group of potentially pathogenic bacteria that underlie these healthcare associated infections (HAIs) that lead to unnecessary discomfort and extended hospitalization.

Bacterial colonization often occurs because of an indirect patient-to-patient transmission due to inadequate hygiene [2]. Depending on the material, bacteria deposited on a surface can survive between 1 and 56 days outside a host organism [3, 4]. Several studies indicate that ambulances may constitute a reservoir of multidrug-resistant bacteria [5–7] and transportation of a patient infected with MRSA has shown an increased risk of environmental contamination after only 10 min [8]. Furthermore, there is a known risk of transferring vancomycin resistant *Enterococcus* (VRE) from patient to environment in the in-hospital setting [9].

Numerous hospitalizations are preceded by a prehospital course. The patients are treated in the ambulance before arriving at the hospital, but evidence of the bacterial contamination level in Europe is almost non-existing, hence calling for an investigation of whether environmental contamination by pathogens also constitutes a challenge in the ambulance service.

The necessity is further emphasized by recent results from a Danish pilot study conducted by the authors that demonstrated pathogenic bacteria, including *S. aureus* and *Enterococcus*, present on prehospital uniforms at the end of a shift [10].

No prior studies have documented the level of contamination in the Danish ambulance environment nor in the Scandinavian, and to the best of our knowledge, have only few recent international studies documented bacterial contamination within the ambulance environment [5–8]. In addition, the comparability of contamination level may be challenged due to variation between different national cleaning guidelines.

An examination of the *S. aureus*/MRSA and *Enterococcus*/VRE prevalence, on cleaned equipment, provides access to state of hygiene in the ambulance service. The dual purpose of this study is to investigate if *S. aureus* and *Enterococcus* are present on newly cleaned blood pressure cuffs, and to uncover type- and resistance patterns of bacteria in the Danish ambulance service.

## Method

This cross-sectional study was conducted in November–December 2014 in cooperation with the “Emergency Medical Services, Capital Region of Denmark”, Copenhagen Fire Brigade and Frederiksberg Fire Brigade in the Capital Region of Denmark, covering 2561 km^2^ with 1,753,976 inhabitants and approximately 125,000 acute frontline courses every year [11].

A total of 47 ambulance emergency responses, and 13 reserve ambulances represented the population from which the sample was drawn. All three prehospital medical services providing the samples, are accredited by The Danish Institute for Quality and Accreditation in Healthcare (IKAS) and the investigation was conducted under the assumption that the ambulance crew are cleaning the blood pressure cuffs in accordance to guidelines.

The UltraCheck NIBP cuffs currently used in the Danish emergency medical service have anti-microbial protection and a fluid barrier built into the fabric that could be considered a hygienic element.

Prior to sampling, the ambulance crew cleaned the cuffs after the end of a patient course, as a part of everyday routine, which implies wiping with ethanol rags (80 %) for approximately 30 s on all surfaces making sure the entire surface is moistened and thoroughly wiped.

Blood pressure cuffs used in the frontline ambulances, regardless of type, age and size were included. Cuffs contaminated to an extent that would lead to exclusion from use in the ambulance service, would have been excluded from the study. However, that situation did not occur and all randomly selected cuffs were included.

Without prior announcement, samples were obtained at five different emergency receptions from 39 different blood pressure cuffs in 39 randomly selected ambulances. Eleven blood pressure cuffs were included twice, with a minimum of two patient courses between sample collections. Thus there were in total 50 samples.

We sampled the inside of the blood pressure cuff (side nearest to patient) using specific 25 cm^2^ agar plates. The imprint was obtained with a pressure of 25 g/cm^2^ for approximately 10 s. Samples were collected in situ minimum 1 min after cleaning, when the cuff appeared dry, and stored at <5 °C until delivered at the laboratory for cultivation and analysis.

### Incubation and identification

In order to determine presence of *S. aureus* we used rapid plasma fibrinogen (RPF) agar plates incubated at 37 ± 1 °C for 24 ± 3 h. For the examination of *Enterococcus*, we used Slanetz agar plates incubated at 44 ± 1 °C for 48 ± 4 h. AnalyTech A/S in Aalborg, Denmark delivered the agar plates used and performed the laboratory investigations presented in Table 1.

**Table 1:** Laboratory investigation

	Agar	Incubation time/temp.
*S. Aureus*	RPF agar	37 °C ± 1 °C: 18–24 h
Resistant analysis	MRSA Select™	37 °C ± 1 °C: 18–24 h
*Enterococcus*	Slanetz agar	44 °C ± 1 °C: 48 ± 4 h

Positive specimens were sent to the national reference laboratory at Statens Serum Institut (SSI) in order to determine type and resistance patterns and genes. *Staphylococcus aureus* isolates were identified by MALDI TOF. Detection of the mecA gene, and subsequent sequencing and analysis of the spa gene was carried out using multiplex PCR. *Enterococcus* isolates were determined for species and resistance (vanA, vanB, vanC) using PCR.

### Data management and statistical analyses

Statistical analyses were performed in STATA13. Presence of *S. aureus* and *Enterococcus* on the 50 agar plates was calculated as proportion (positive or negative) and when positive expressed by CFU per 25 cm^2^ including mean, SD, and range.

### Ethical considerations

The study was exempted from ethical approval since it involved neither humans nor animals. Collection of data was carried out with respect towards the pre-hospital patients in the Capital Region and all samples were obtained *after* delivering the patient to the ward.

## Results

*Staphylococcus aureus* and *Enterococcus* were present on the blood pressure cuffs; however, to a limited extent. Ten percent of 50 imprints were found positive for *S. aureus* and the average level of contamination was 0.52 CFU per 25 cm^2^ (SD 1.87). The minimum and maximum values ranged between 0 and 11 CFU per 25 cm^2^. All *S. aureus* isolates were methicillin susceptible Table 2.

**Table 2:** Level of contamination, *n* = *50*

	*S. Aureus*	*Enterococcus*
Mean CFU per 25 cm^2^	0.52	0.06
SD	1.87	0.42
Min–max CFU per 25 cm^2^	0–11	0–3
Number (%)	5 (10)	1 (2)

Two percent of 50 imprints were found positive for *Enterococcus* and the average level of contamination was 0.06 CFU per 25 cm^2^ (SD 0.42). The minimum and maximum values ranged from 0 to 3 CFU per 25 cm^2^. The isolates determined to be *Enterococcus faecalis*, were susceptible to vancomycin.

## Discussion

The dual purpose of this pilot study was to demonstrate the presence of *S. aureus* and *Enterococcus* on newly cleaned blood pressure cuffs and to display prevalence of MSSA/MRSA and VRE.

We found *S. aureus* and *Enterococcus* present on the presumed clean blood pressure cuffs, albeit to a very limited extent and we found no MRSA or VRE. Our results differ significantly from findings in American ambulance services which indicates a more extensive presence of MRSA [6, 7]. The fact that we did not find MRSA may be explained by a generally low prevalence of MRSA in Denmark, due to a profound focus on prevention of multi resistant bacteria supported by a comprehensive surveillance system [12].

Presence of faecal bacteria on clean blood pressure cuffs suggests cross-contamination, a trend also seen in a study by Nigam and Cutter who found *S. aureus* and coliform bacteria in the ambulance environment after cleaning [13]. Unfortunately, Nigam and Cutter did not include susceptibility analyses in their study, so any further comparison is not possible.

When interpreting our results, one must consider the fact that infection control in the prehospital setting faces numerous challenges. First of all the prehospital setting is accelerating with courses around the clock, with one patient replacing the other. In addition, the ambulance crew encounters a wide variety of environment, for example home environments, highway environments, agricultural environments and nursing homes that makes it highly unlikely to achieve ambulances completely free of pathogens. Nevertheless, routine cleaning of ambulances and equipment in-between patient courses in order to reduce bacterial contamination is, and should be, prioritized.

According to the *Spaulding Classification System,* blood pressure cuffs are considered non-critical patient care items with limited risk of infection when in contact with intact skin. Nevertheless, the apparent cross-contamination may be associated with lack of hygiene- or cleaning compliance among the crew and have led to an evaluation of current cleaning procedures and possible need for future training of the ambulance crew. We have adopted the acceptance levels after cleaning for hand touch sites similar to levels within the hospital, which means that pathogens are not accepted [14].

Our study has some limitations. First, our sample size was relatively small and limited to one geographical area, which challenges a generalisation to other areas in Denmark. Second, we did not use agar with neutralizers despite testing newly cleaned and disinfected cuffs, which could lead to an underestimation of the bacteria to be found. In addition, we did not test the cleaning method using an interventional design neither did we investigate the correlation between level of contamination and individual cleaning performance, hence making an evaluation of the cleaning procedure and crew compliance impossible.

It would also have been useful to test the sensitivity of the method in order to evaluate the precision of our results. However, the primary purpose of this pilot study was to demonstrate a presence of potential pathogenic and resistant bacteria on supposed clean prehospital equipment, hence documenting bacterial contamination and need for further focus on prehospital hygiene.

Overall, there seems to be a lack of evidence internationally regarding prehospital hygiene and bacterial contamination, hence making it difficult to conclude on the extent of the infection risk associated with the prehospital course, but studies indicate that—in hospital—environmental contamination makes an important contribution to infection [15, 16].

## Conclusion

*Staphylococcus aureus* and *Enterococcus* were detectable on equipment thought to be clean. However, all detected bacteria showed susceptibility towards methicillin or vancomycin. Findings of pathogens after cleaning may be due to cross-contamination, improper cleaning and limited effect of the currently used cleaning procedure and are thought to affect the risk of infection. Therefore, we recommend a thorough evaluation of current cleaning procedures as well as increased focus on and further research into hygiene challenges in a prehospital setting. Future studies should be performed in order to demonstrate the level of bacterial contamination in all areas of the medical service, e.g., the ambulance environment, medical equipment, staff uniform and hand hygiene. Furthermore, in order to establish evidence for different cleaning procedures in situ we recommend testing the effect of different cleaning interventions by interventional designs.
